# The prevalence of rheumatoid arthritis in Western Australia

**DOI:** 10.1186/s41927-022-00324-5

**Published:** 2022-12-31

**Authors:** Khalid Almutairi, Charles Inderjeeth, David B Preen, Helen Keen, Johannes Nossent

**Affiliations:** 1 Faculty of Health and Medical Sciences, School of Medicine, The University of Western Australia, 35 Stirling Highway, Perth, WA 6009 Australia; 2 King Fahd Specialist Hospital, Burydah, Saudi Arabia; 3 Sir Charles Gairdner and Osborne Park Health Care Group, Perth, Australia; 4 Faculty of Health and Medical Sciences, School of Population and Global Health, University of Western Australia, Perth, WA Australia; 5 Fiona Stanley Hospital, Murdoch, WA Australia

**Keywords:** Prevalence, Arthritis, Rheumatoid, Hospitalisation, Associated factors, Biological therapy

## Abstract

**Background:**

Rheumatoid arthritis (RA) is the most common form of autoimmune arthritis, but the prevalence in Australia is unknown. We estimated RA period prevalence and identified factors associated with frequent RA hospitalisations, using linked administrative health and state-specific Australian Pharmaceutical Benefits Scheme (PBS) datasets in Western Australia (WA) from 1995 to 2014.

**Methods:**

This was a longitudinal population-based cohort study using two independent datasets to identify prevalent RA patients. RA prevalence was calculated per 1000 hospital separations and biological therapy users. RA patients were identified in the WA linked health dataset using ICD codes 714.0–714.9 and M05.00–M06.99. Dispensing data on biological therapy for RA were obtained from PBS records and converted to defined daily doses /1000 population/day. Multivariable logistic regression was used to analyse factors associated with frequent RA hospitalisations (> 2/year), controlling for sex, age, and geographic locations. Potential interactions were assessed using logistic regression in a stepwise approach.

**Results:**

A total of 17,125 RA patients had 50,353 hospital separations between 1995 and 2014, averaging three hospitalisations per patient over 20 years. The RA period prevalence was 3.4 per 1000 separations (0.34%; 95% CI 0.33–0.34), while the RA period prevalence based on biological therapy use was 0.36% (95% CI 0.35–0.37). The corrected RA prevalence based on biological therapy usage was 0.36% (95% CI 0.35–0.37) for the 2005–2009 and increased to 0.72% (95% CI 0.70–0.74) in 2010–2014 period. Associated factors for frequent RA hospitalisations were being female [1.21 (95% CI 1.15–1.26)], aged 60–69 years [4.45 (95% CI 3.74–5.30)], living in rural areas [1.12 (95% CI 1.02–1.24)]. The odd ratio of interaction between these associated factors was 1.34 (95% CI 1.16–1.55).

**Conclusion:**

The minimal prevalence of RA in Western Australia is 0.34–0.36%, which falls within the literature range. Older female RA patients in rural were more likely to be hospitalised, suggesting unmet primary care for needs.

**Supplementary Information:**

The online version contains supplementary material available at 10.1186/s41927-022-00324-5.

## Key points

- The study estimated a minimum prevalence of Rheumatoid Arthritis of 0.34% in Western Australia based on hospital diagnosis records over 20 years and 0.36% based on biological therapy dispensing over a decade.
- The study found that older females with Rheumatoid Arthritis who live in rural areas are more likely to experience frequent hospitalisations suggesting unmet needs in their primary care access.

**Supplementary Information:**

The online version contains supplementary material available at 10.1186/s41927-022-00324-5.

## Background

Rheumatoid arthritis (RA) is a heterogeneous chronic autoimmune disease that affects the synovial joint lining and may result in permanent joint destruction, premature death, and socioeconomic burden [1]. RA is deemed a multifactorial disorder in which numerous hereditary and environmental factors affect the disease prevalence [2]. The global prevalence of RA is estimated to be 0.46% in population-based studies [3], but there is a paucity of data from Australia. The prevalence of RA in the Australian population has been estimated at 1.9%, based on self-reported data as part of the National Health Survey (2017–2018) [4]. This would make Australia the country with the world’s highest reported RA prevalence [5], but self-reported diagnosis is unreliable for identifying RA cases as only a low proportion (21–34%) will have RA verified upon clinical review [6].

Although RA is one of Australia's national health priority areas and gathering information about the RA burden of disease was one of the national action plans from 2004 to 2006 [7], epidemiological studies on RA are scarce. Prevalence data have become particularly essential in recent years as more treatment options have become available [1] and can offers a framework for predicting present and growing health care service requirements [8]. Information on RA prevalence at local, national and international levels through population-based studies provides insight on patient outcomes, health care utilization, and related costs needed for evidenced-based policy responses [9]. Our recent meta-analysis confirmed that data on RA prevalence in Australia are very limited and Western Australia figures may serve as a proxy for national data [3]. Linked administrative health data can provide a timely and cost-effective approach for epidemiological study in the Australian population [10]. Also, the use of medication prescriptions database provides a reliable prevalence estimate of several chronic diseases, including rheumatologic conditions in the general population [11, 12].

We undertook the present study to describe the prevalence of RA based on two independent data sources namely linked hospital admission data and biologic disease-modifying anti-rheumatic drugs (bDMARDs) usage by RA patients in Western Australia (WA) between 1995 and 2014. This study also analysed factors associated with frequent RA hospitalisations.

## Methods

### Study design

This was a longitudinal population-based cohort study using two independent datasets to identify prevalent RA patients. This included 1) a statewide linked administrative health hospital-based datasets over the period 1995–2014 and 2) a state-specific Australian Pharmaceutical Benefits Scheme (PBS) dataset on usage of biological DMARD (b-DMARDS) for RA in WA from 2003 to 2014. We followed the STrengthening the Reporting of OBservational studies in Epidemiology (STROBE) guidelines [13] to ensure the accuracy and completeness of reporting (Additional file 1: Table S1).

### Data sources

The Western Australian Rheumatic Disease Epidemiological Registry (WARDER) [14, 15] contains deidentified longitudinally linked health data for all hospital-ascertained RA patients in the WA Hospital Morbidity Data Collection, Emergency Data Collection, and the WA Death Registry. WARDER data were extracted and linked by the WA Data Linkage Branch through probabilistic matching with clerical review based on patient's name, date of birth, sex and residential address with high linkage accuracy (99.9%) [16]. RA patients were identified in the WARDER as those patients with a hospital separation listing a physician-based discharge primary or secondary diagnosis of RA using the International Classification of Diseases (ICD) codes for RA (ICD-9-AM for the period from January 1995 to June 1999, and ICD-10-AM for the period July 1999 to June 2015). The list of diagnostic codes used for extraction were verified by the Western Australian clinical coding authority in the WA Department of Health and shown in Additional file 1: Table S2. RA patients were excluded if they had at least two consecutive health contacts for other different types of arthritis, such as psoriatic arthritis, ankylosing spondylitis, other spondyloarthropathies or connective tissue disorders [17]. This method has shown a higher than 90% positive predictive value (PPV) for identifying RA patients based on rheumatologist‐reported diagnosis as a reference standard [18].

The PBS has subsidised bDMARD therapy for RA since 2003. De-identified dispensing data of bDMARDs was obtained from the public domain based on PBS codes for authority required scripts, that are specific for RA and require that the diagnosis is made by a rheumatologist [19]. Dispensing date were converted to defined daily doses (DDD)/1000 population/day using the WA general population census [20]. The DDD for each drug was identified from the World Health Organisation (WHO) for the 2021 ATC/DDD Index [21]. If no detail on the DDD value was available, the value was determined based on the recommended daily dose for RA bDMARDs in Australia [22].

### Statistical methods

Descriptive statistics were determined for all patient characteristics, with results presented as mean and standard deviation for continuous variables and counts and proportions for categorical variables.

We estimated RA period-prevalence rates per 1,000 hospital separations and annual average percentage changes, with the total number of hospital separations each year and five-year period (1995–1999, 2000–2004, 2005–2009, 2010–2014). Each year, the total number of hospital separations is obtained from the Australian Institute of Health and Welfare (AIHW) domain. We also used bDMARDs utilisation to estimate the period-prevalence of RA in the WA population between 2003 and 2014. bDMARDs utilisation was measured as the total number of DDD/1000 population/day each year of the study period.

Period-prevalence of RA was calculated as the number of patients who used standard doses daily of RA bDMARDs treatment during a five-year period (2005–2009 and 2010–2014), divided by the WA general population at the period midpoint. We assumed that the proportion of patients who used bDMARDs reflects 50% of RA in the community based on published literature [23, 24], as no adequate utilisation studies are available in Australia. Thus, RA prevalence was also corrected by a factor of 2 (100/50) to estimate RA prevalence in WA general population from these data.

Frequent RA hospital separation was defined as two or more separations for RA diagnostic codes (primary or secondary) in a calendar year [25]. The potential associated factors assessed were sex, age, and geographic locations as commonly published in the literature [5, 26, 27]. The Accessibility/Remoteness Index of Australia (ARIA) is an index of remoteness derived from road distance measures between populated localities and service centres. Using patient's baseline residential postcode, they were assigned to three geographical population settings: urban (major cities), rural (inner and outer regional) and remote areas (remote and very remote areas). We used the multivariable logistic regression to identify factors associated with frequent RA hospital separations as a binomial outcome. Each model was controlled for sex, age, and geographic locations. Potential interactions were assessed using logistic regression in a backward stepwise selection approach. Adjusted odds ratios (OR) and 95% confidence intervals (CI) were calculated. Unfortunately, PBS data was limited to the utilisation use of biological treatments based on DDD. The PBS data do not contain any covariates (sex, gender, or geographical location) to perform regression models. The joinpoint regression method was used to understand the significant changes in the prevalence of RA over time. All statistical analyses were performed in R version 4.0.2, with statistical significance set at two-tailed with the alpha level of 0.05.

### Ethics approval

Ethic approval of de-identified data of this study was granted by the WA Department of Health Human Research Ethics Committee (approval number 2016/24) and the Human Research Ethics Committee of the University of Western Australia (approval number RA/4/20/4070). The ethics approval letter can be found in Additional file 1: Appendix A.

## Results

### Period-prevalence of RA—hospital data

**Fig. 1:**
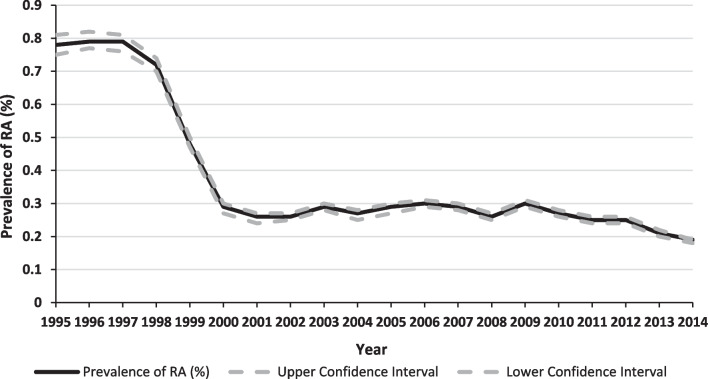
The prevalence of rheumatoid arthritis in Western Australia extrapolated from hospitalisations over time. RA = rheumatoid arthritis

Over the study period, the total number of hospital separations in WA was 14,936,094. When combined with the total number of hospital separations for RA patients (50,353), this produces an RA prevalence of 0.34% (3.4 per 1000 hospital separations). The estimated period-prevalence of RA was 0.7%, 0.27%, 0.28%, and 0.23% for the time periods 1995–1999, 2000–2004, 2005–2009, and 2010–2014 respectively (Fig. 1).

Among all 17,125 patients (mean age was 62.8 ± 16.1 years, 73% female) hospitalisation rates (average of three hospitalisations over 20 years) were higher for females than males (Additional file 1: Table S3). Most RA patients lived in urban areas (78.0%), 16% lived in rural areas, and 6.1% lived in remote areas. The total length of stays was 343,128 days over the study period (Additional file 1: Table S4). The mean length of stay in females dropped from 11 days (95% CI 14.27–7.66) in 1995 to 3.75 days (95% CI 4.14–3.35) in 2014. For males, the mean length of stay declined from 8 days (95% CI 8.65–7.38) in 1995 to 5.23 days (95% CI 6.48–3.97) in 2014.

Multivariable logistic regression analysis to identify factors associated with frequent hospitalisations in RA patients (Table 1) found increased odds for females (OR 1.21, 95% CI 1.15–1.26, *P* < 0.01), patients in the age group between 60 and 69 years (OR 4.45, 95% CI 3.74–5.30, *P* < 0.01), and rural residents (OR 1.12, 95% CI 1.02–1.24, *P* < 0.01), compared with the reference category (males, patients aged less than 19 years, and remote areas).

**Table 1:** Patients related characteristics and adjusted associated factors with frequent hospital separations for rheumatoid arthritis patients in Western Australia (1995–2014)

Characteristics	n (%)	Frequent hospitalisations (%)	Non frequent hospitalisations (%)	OR (95% CI)	Adjusted OR (95% CI)†	Wald test (*P*-value)
*Sex*						
Male	13,798 (27.4%)	10,298 (74.6%)	3500 (25.4%)	1	1	–
Female	36,555 (72.6%)	28,427 (77.8%)	8128 (22.2%)	1.18 (1.13–1.24)	1.21 (1.15–1.26)	.01
*Age groups*						
≤ 19**‡**	546 (1.1%)	254 (46.5%)	292 (53.5%)	1	1	–
20–29	1,334 (2.6%)	945 (70.8%)	389 (29.2%)	2.79 (2.27–3.43)	2.74 (2.23–3.37)	< .01
30–44	5,373 (10.7%)	4,055 (75.5%)	1318 (24.5%)	3.53 (2.95–4.23)	3.52 (2.94–4.21)	< .01
45–59	13,269 (26.4%)	10,541 (79.4%)	2728 (20.6%)	4.44 (3.73–5.28)	4.45 (3.74–5.29)	< .01
60–69	11,815 (23.4%)	9,381 (79.4%)	2434 (20.6%)	4.43 (3.72–5.27)	4.45 (3.74–5.30)	< .01
70–84	15,361 (30.5%)	11,620 (75.6%)	3741 (24.4%)	3.57 (3.00–4.24)	3.55 (2.99–4.22)	< .01
85+	2655 (5.3%)	1929 (72.7%)	726 (27.3%)	3.05 (2.53–3.68)	2.97 (2.46–3.59)	< .01
*ARIA classification*						
Remote	3053 (6.1%)	2303 (75.4%)	750 (24.6%)	1	1	–
Rural	8065 (16.0%)	6,223 (77.2%)	1842 (22.8%)	1.10 (0.99–1.21)	1.12 (1.02–1.24)	.01
Major cities	39,235 (77.9%)	30,199 (77%)	9036 (23%)	1.08 (0.99–1.18)	1.11 (1.01–1.21)	.01

*ARIA* Accessibility/Remoteness Index of Australia; *CI* confidence intervals; *OR* odds ratios; *RA* rheumatoid arthritis

†Adjusted for age, sex, and geographic locations

**‡**The age group included juvenile rheumatoid arthritis

The odd ratio of interaction between these associated factors was 1.34 (95% CI 1.16–1.55) and interaction analysis can be found in the Additional file 1: Table S5. The joinpoint regression analysis identified a breakpoint in 2001 at which statistically significant changes occurred in RA prevalence over time (Additional file 1: Fig. S1).

### Period-prevalence of RA–PBS data

**Fig. 2:**
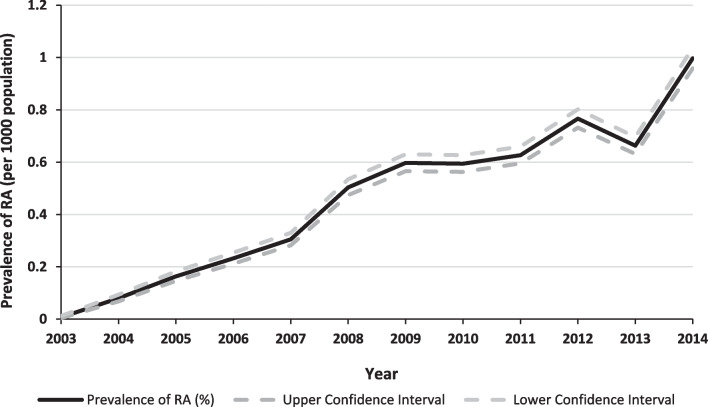
The prevalence of rheumatoid arthritis in Western Australia extrapolated from biological therapy usage data over time. RA = rheumatoid arthritis

Total (DDD/1000 population/day) for RA and the number of RA patients that use the standard daily dose of RA bDMARDs in WA over the study period are listed in Additional file 1: Table S6 and Fig. 2.

Based on these data, the estimated period-prevalence of RA was 0.18% for 2005–2009 and rose to 0.36% for 2010–2014 (Additional file 1: Fig. S2). The annual average change increased by 60.2% in RA patients who used the DDD of RA bDMARDs since 2003 (Additional file 1: Table S6). The corrected RA prevalence based on bDMARDs usage was 0.36% and 0.72% for the 2005–2009 and 2010–2014 periods, respectively.

## Discussion

This study found the minimum prevalence of RA in WA to be 0.34% based on hospital diagnosis records over 20 years and 0.36% based on bDMARDs dispensing over a decade. These estimates align with the international literature [26, 28], but are well below the 1.9% prevalence reported by national agencies such as the AIHW [4]. The current study findings indicate that self-reported RA data in Australia significantly overestimate the prevalence of RA [6], consistent with confirmed (0.27%) versus self-reported prevalence (1.9%) data from South Korea [28].

The estimates of RA prevalence in this study should be considered minimum prevalence rates as they are based on two separate population-based datasets. RA patients who were never admitted to hospital may have been missed, but in the literature, the mean number of hospital admissions for RA patients for disease complications or comorbidity varies from 0.13 to 0.34 per year [29], which lies within the range of the current study's annual RA hospital separations (0.15%). This suggests that the long-term observation in this study covered the large majority of RA patients state-wide.

Also, the study found no evidence that RA separations were over-represented in the hospital data, as RA separations were less than separations for other conditions. Similarly, not all RA patients need to progress to bDMARD and access to subsidised biological therapy in Australia is restricted to patients with severe or unresponsive disease as assessed by the treating rheumatologist. Based on scarce literature data and personal experience, we assumed that this regards 50% of all RA patients in WA and accordingly corrected the RA period prevalence which fell within the range of prevalence estimates derived from Australian general practice data [30].

Our data are also consistent with findings by the South Swedish Arthritis group [31], where RA prevalence based on a rheumatologist‐reported diagnosis and the use biological therapies was 0.66% [31]. Given the rapid expansion of biological therapies since 2003, not surprising that the corrected period-prevalence estimate based on bDMARDs utilisation in our study rose to 0.72% by 2014. This agrees with our previous study on diagnostic accuracy of RA [18], where adding the biological codes further improved case ascertainment based on rheumatologist‐reported diagnosis [18]. Similarly, in the Korean study, the addition of bDMARD codes in the RA case definition identified RA patients with a high degree of accuracy (PPV 97.2%) [32].

In our study, the rapid increase of bDMARDs prevalence from 0.36% by 2009 to 0.72% by 2014 has important implications for health care utilisation and increase costs [33], although most of these effects are attributable to early and aggressive therapy in combination with conventional DMARDs in terms of disease control and achieve remission for a large proportion of RA patients. This can be seen clearly in context of falling level in hospital admission for RA based on increase of bDMARDs utilisation, which is a complete turnaround of the earlier therapeutic pyramid [34, 35].

Finally, we identified females aged 60–69 as the group with the highest admission rates, which is consistent with literature [28, 36, 37]. Our data suggests that living in rural areas of WA adds to the possibility of being frequently hospitalised for RA, which could indicate limited local access to primary health care [38]. Based on literature, postulated reasons for frequent hospitalisations of RA older females in rural areas include rheumatologists’ shortage [38], delay in earlier diagnosis and treatment of RA [39], and a lower socioeconomic status of the rural population [40].

The study’s main strength is the availability of validated whole-population linked health data for a 20 years observation period and PBS based dispensing data over 10 years, which allowed an estimate of RA prevalence from two separate sources, but both using a physician-confirmed RA diagnosis. This resulted in a sufficient sample size and power to estimate the prevalence in WA and the generalisability findings to estimate and compare RA prevalence with other Australian states.

There are some limitations to this study. The case definition of RA included all ages and therefore included juvenile rheumatoid arthritis, which is different from adult RA in symptoms, remission period and disability [41]. However, the study added this age group as an age group reference for RA cases and also bDMARDs have been used to treat Juvenile RA since 2003 [42]. RA patients who were not admitted to the hospital over the 20-year study period have not been included in the hospital data.

Also, as not all RA patients require bDMARD therapy, we corrected the bDMARD-based RA prevalence according to the best available evidence. We assumed that the proportion of patients who used bDMARDs reflects 50% of RA in the community based on published literature [23, 24], as no adequate utilisation studies are available in Australia. This may either be an underestimate or overestimate without more reliable data.

## Conclusion

The estimated prevalence of RA in Western Australia over the study period was 0.35%, which is in line with international data but well below the figure currently employed by national organisations in Australia. The study found that older females with RA who live in rural areas are more likely to experience frequent hospitalisations suggesting unmet needs in their primary care access.

## Supplementary Information

**Additional file 1**. **Table S1:** STROBE Statement checklist of items that should be included in reports of observational studies. **Table S2:** A list of ICD 9-AM and ICD 10-AM diagnosis codes for RA. **Table S3:** Hospital separations for Rheumatoid Arthritis in Western Australia hospitals (1995-2014). **Table S4:** The mean and confidence interval of rheumatoid arthritis patients' length of stays in Western Australian hospitals (1995–2014). **Table S5:** Odd interaction ratio between female rheumatoid arthritis patients who were 60-69 years old and lived in rural areas. **Figure S1:** Joinpoint regression model visualisation. **Figure S2:** The prevalence of Rheumatoid Arthritis percentage in Western Australia extrapolated from biological therapy usage data over time. **Table S6:** Total RA bDMARDs utilisation (DDD/1000 population/day) and number RA patients use standard dose daily (DDD) of RA bDMARDs at WA, 1995–2014. **Appendix A:** Ethics approval letter.

## Data Availability

All data generated or analysed is available in this article or the Additional file 1.

## References

[1] Guo Q, Wang Y, Xu D, Nossent J, Pavlos NJ, Xu J (2018). Rheumatoid arthritis: pathological mechanisms and modern pharmacologic therapies. Bone Res.

[2] Smolen JS, Aletaha D, Barton A, Burmester GR, Emery P, Firestein GS, Kavanaugh A, McInnes IB, Solomon DH, Strand V, Yamamoto K (2018). Rheumatoid arthritis. Nat Rev Dis Prim.

[3] Almutairi K, Nossent J, Preen D, Keen H, Inderjeeth C (2021). The global prevalence of rheumatoid arthritis: a meta-analysis based on a systematic review. Rheumatol Int.

[4] Australian Institute of Health and Welfare. Rheumatoid arthritis 2020. https://www.aihw.gov.au/reports/chronic-musculoskeletal-conditions/rheumatoid-arthritis/contents/who-gets-rheumatoid-arthritis.

[5] Shapira Y, Agmon-Levin N, Shoenfeld Y (2010). Geoepidemiology of autoimmune rheumatic diseases. Nat Rev Rheumatol.

[6] Al Mutairi KB, Nossent JC, Inderjeeth CA (2020). Validity of self-reported diagnosis of rheumatoid arthritis. J Rheumatol.

[7] Australian Institute of Health and Welfare. National indicators for monitoring osteoarthritis, rheumatoid arthritis and osteoporosis 2006. p. 55. https://www.aihw.gov.au/getmedia/ceb67de7-3497-472b-9bbc-50477eac32af/nimorao.pdf.aspx?inline=true.

[8] Hanly JG, Thompson K, Skedgel C (2015). The use of administrative health care databases to identify patients with rheumatoid arthritis. Open Access Rheumatol.

[9] Fox DM (2005). Evidence of evidence-based health policy: the politics of systematic reviews in coverage decisions. Health Aff.

[10] Sibthorpe B, Kliewer E, Smith L (1995). Record linkage in Australian epidemiological research: health benefits, privacy safeguards and future potential. Aust N Z J Public Health.

[11] Chini F, Pezzotti P, Orzella L, Borgia P, Guasticchi G (2011). Can we use the pharmacy data to estimate the prevalence of chronic conditions? A comparison of multiple data sources. BMC Public Health.

[12] O'Dell JR (2004). Therapeutic strategies for rheumatoid arthritis. N Engl J Med.

[13] von Elm E, Altman DG, Egger M, Pocock SJ, Gøtzsche PC, Vandenbroucke JP (2007). The Strengthening the Reporting of Observational Studies in Epidemiology (STROBE) statement: guidelines for reporting observational studies. Lancet.

[14] Nossent JC, Raymond W, Keen H, Preen DB, Inderjeeth CA (2020). Infection rates before and after diagnosis of IgA vasculitis in childhood: a population-wide study using non-exposed matched controls. J Rheumatol.

[15] Ognjenovic M, Raymond W, Inderjeeth C, Keen H, Preen D, Nossent J (2020). The risk and consequences of vertebral fracture in patients with ankylosing spondylitis: a population-based data linkage study. J Rheumatol.

[16] Kelman CW, Bass AJ, Holman CD (2002). Research use of linked health data—a best practice protocol. Aust N Z J Public Health.

[17] Lacaille D, Anis AH, Guh DP, Esdaile JM (2005). Gaps in care for rheumatoid arthritis: a population study. Arthritis Rheum.

[18] Almutairi K, Inderjeeth C, Preen DB, Keen H, Rogers K, Nossent J (2021). The accuracy of administrative health data for identifying patients with rheumatoid arthritis: a retrospective validation study using medical records in Western Australia. Rheumatol Int.

[19] Medicare Australia. Pharmaceutical benefits schedule item reports 2020. http://medicarestatistics.humanservices.gov.au/statistics/pbs_item.jsp.

[20] Australian Bureau of Statistics. Population by age and sex 2014. http://stat.data.abs.gov.au/Index.aspx?DataSetCode=ABS_ERP_ASGS2016.

[21] World Health Organisation. ATC/DDD Index 2021. https://www.whocc.no/atc_ddd_index/.

[22] Simone R (2021). Australian medicines handbook.

[23] Allaire S, Wolfe F, Niu J, Zhang Y, Zhang B, LaValley M (2008). Evaluation of the effect of anti-tumor necrosis factor agent use on rheumatoid arthritis work disability: the jury is still out. Arthritis Rheum.

[24] Sergeant JC, Hyrich KL, Anderson J, Kopec-Harding K, Hope HF, Symmons DPM, Co-Investigators R, Barton A, Verstappen SMM (2018). Prediction of primary non-response to methotrexate therapy using demographic, clinical and psychosocial variables: results from the UK Rheumatoid Arthritis Medication Study (RAMS). Arthritis Res Ther.

[25] Ciocci A, Buratti L, Coari G, Di Franco M, Iagnocco AM, Mauceri MT, Serio A (2001). Rheumatoid arthritis: frequency of hospitalization and evaluation of economic burden. Reumatismo.

[26] Almutairi K, Nossent J, Preen D, Keen H, Inderjeeth C (2021). The global prevalence of rheumatoid arthritis: a meta-analysis based on a systematic review. Rheumatol Int.

[27] Tobon GJ, Youinou P, Saraux A (2010). The environment, geo-epidemiology, and autoimmune disease: rheumatoid arthritis. J Autoimmun.

[28] Sung YK, Cho SK, Choi CB, Bae SC (2013). Prevalence and incidence of rheumatoid arthritis in South Korea. Rheumatol Int.

[29] Rat AC, Boissier MC (2004). Rheumatoid arthritis: direct and indirect costs. Jt Bone Spine.

[30] Harrison C, Henderson J, Miller G, Britt H (2017). The prevalence of diagnosed chronic conditions and multimorbidity in Australia: a method for estimating population prevalence from general practice patient encounter data. PLoS ONE.

[31] Englund M, Jöud A, Geborek P, Felson DT, Jacobsson LT, Petersson IF (2010). Prevalence and incidence of rheumatoid arthritis in southern Sweden 2008 and their relation to prescribed biologics. Rheumatology.

[32] Cho SK, Sung YK, Choi CB, Kwon JM, Lee EK, Bae SC (2013). Development of an algorithm for identifying rheumatoid arthritis in the Korean National Health Insurance claims database. Rheumatol Int.

[33] Almutairi K, Nossent J, Preen DB, Keen H, Inderjeeth C (2021). The temporal association between hospital admissions, biological therapy usage and direct health care costs in rheumatoid arthritis patients. Rheumatol Int.

[34] Smolen JS, Landewé RB, Bijlsma JW, Burmester GR, Dougados M, Kerschbaumer A, McInnes IB, Sepriano A, Van Vollenhoven RF, De Wit M. EULAR recommendations for the management of rheumatoid arthritis with synthetic and biological disease-modifying antirheumatic drugs 2020: 2019 update. 10.1136/annrheumdis-2019-216655 31969328

[35] Wilske KR, Healey LA (1990). Challenging the therapeutic pyramid: a new look at treatment strategies for rheumatoid arthritis. J Rheumatol Suppl.

[36] Rai SK, Avina-Zubieta JA, McCormick N, De Vera MA, Lacaille D, Sayre EC, Choi HK (2017). Trends in gout and rheumatoid arthritis hospitalizations in Canada from 2000 to 2011. Arthritis Care Res (Hoboken).

[37] Finckh A, Gilbert B, Hodkinson B, Bae SC, Thomas R, Deane KD, Alpizar-Rodriguez D, Lauper K (2022). Global epidemiology of rheumatoid arthritis. Nat Rev Rheumatol.

[38] Roberts LJ, Lamont EG, Lim I, Sabesan S, Barrett C (2012). Telerheumatology: an idea whose time has come. Intern Med J.

[39] National Rural Health Alliance. Poverty in rural & remote Australia 2017. https://www.ruralhealth.org.au/sites/default/files/publications/nrha-factsheet-povertynov2017.pdf.

[40] Putrik P, Ramiro S, Keszei AP, Hmamouchi I, Dougados M, Uhlig T, Kvien TK, Boonen A (2016). Lower education and living in countries with lower wealth are associated with higher disease activity in rheumatoid arthritis: results from the multinational COMORA study. Ann Rheum Dis.

[41] Mehta J. Juvenile Idiopathic Arthritis (JIA)—Pediatrics—MSD Manual Professional Edition 2020. https://www.msdmanuals.com/en-au/professional/pediatrics/juvenile-idiopathic-arthritis/juvenile-idiopathic-arthritis-jia?query=Juvenile%20Idiopathic%20Arthritis%20(JIA).

[42] Australian Institute of Health and Welfare. A snapshot of juvenile arthritis 2013. https://www.aihw.gov.au/getmedia/125411fa-7c84-4753-aec1-0a0d76a63a2d/14900.pdf.aspx?inline=true#:~:text=Etanercept%20and%20adalimumab%20are%20biologic,and%20adalimumab%20since%20November%202010.

