# Supplementary material for: The prevalence of rheumatoid arthritis in Western Australia
Source: BMC Rheumatol. 2022 Dec 31;6:93. doi: 10.1186/s41927-022-00324-5 (PMC9804946; doi:10.1186/s41927-022-00324-5)

**Additional file 1:**

**Table S1: STROBE Statement checklist of items that should be included in reports of observational studies.**

|  | Item No | Recommendation | Page  No |
| --- | --- | --- | --- |
| Title and abstract | 1 | (*a*) Indicate the study’s design with a commonly used term in the title or the abstract | 2 |
|  |  | (*b*) Provide in the abstract an informative and balanced summary of what was done and what was found | 2 |
| Introduction | | | |
| Background/rationale | 2 | Explain the scientific background and rationale for the investigation being reported | 3-4 |
| Objectives | 3 | State specific objectives, including any prespecified hypotheses | 4 |
| Methods | | | |
| Study design | 4 | Present key elements of study design early in the paper | 4 |
| Setting | 5 | Describe the setting, locations, and relevant dates, including periods of recruitment, exposure, follow-up, and data collection | 4-5 |
| Participants | 6 | (*a*) *Cohort study*—Give the eligibility criteria, and the sources and methods of selection of participants. Describe methods of follow-up  *Case-control study*—Give the eligibility criteria, and the sources and methods of case ascertainment and control selection. Give the rationale for the choice of cases and controls  *Cross-sectional study*—Give the eligibility criteria, and the sources and methods of selection of participants | 4-5 |
|  |  | (*b*) *Cohort study*—For matched studies, give matching criteria and number of exposed and unexposed  *Case-control study*—For matched studies, give matching criteria and the number of controls per case | 4-5 |
| Variables | 7 | Clearly define all outcomes, exposures, predictors, potential confounders, and effect modifiers. Give diagnostic criteria, if applicable | 6-7 |
| Data sources/ measurement | 8* | For each variable of interest, give sources of data and details of methods of assessment (measurement). Describe comparability of assessment methods if there is more than one group | *4-5* |
| Bias | 9 | Describe any efforts to address potential sources of bias | 11 |
| Study size | 10 | Explain how the study size was arrived at | 7 |
| Quantitative variables | 11 | Explain how quantitative variables were handled in the analyses. If applicable, describe which groupings were chosen and why | 6-7 |
| Statistical methods | 12 | (*a*) Describe all statistical methods, including those used to control for confounding | 6-7 |
|  |  | (*b*) Describe any methods used to examine subgroups and interactions | Table 3 |
|  |  | (*c*) Explain how missing data were addressed | NA |
|  |  | (*d*) *Cohort study*—If applicable, explain how loss to follow-up was addressed  *Case-control study*—If applicable, explain how matching of cases and controls was addressed  *Cross-sectional study*—If applicable, describe analytical methods taking account of sampling strategy | NA |
|  |  | (*e*) Describe any sensitivity analyses | NA |

| Results | | | |
| --- | --- | --- | --- |
| Participants | 13* | (a) Report numbers of individuals at each stage of study—eg numbers potentially eligible, examined for eligibility, confirmed eligible, included in the study, completing follow-up, and analysed | Table 4 |
|  |  | (b) Give reasons for non-participation at each stage | NA |
|  |  | (c) Consider use of a flow diagram | NA |
| Descriptive data | 14* | (a) Give characteristics of study participants (eg demographic, clinical, social) and information on exposures and potential confounders | Table 3 |
|  |  | (b) Indicate number of participants with missing data for each variable of interest | NA |
|  |  | (c) *Cohort study*—Summarise follow-up time (eg, average and total amount) | 7-8 |
| Outcome data | 15* | *Cohort study*—Report numbers of outcome events or summary measures over time | *Table 1 &3&4* |
|  |  | *Case-control study—*Report numbers in each exposure category, or summary measures of exposure | *NA* |
|  |  | *Cross-sectional study—*Report numbers of outcome events or summary measures | *NA* |
| Main results | 16 | (*a*) Give unadjusted estimates and, if applicable, confounder-adjusted estimates and their precision (eg, 95% confidence interval). Make clear which confounders were adjusted for and why they were included | Table 3 |
|  |  | (*b*) Report category boundaries when continuous variables were categorized | Table 2 & 3 |
|  |  | (*c*) If relevant, consider translating estimates of relative risk into absolute risk for a meaningful time period | Table 3 |
| Other analyses | 17 | Report other analyses done—eg analyses of subgroups and interactions, and sensitivity analyses | Table 3 |
| Discussion | | | |
| Key results | 18 | Summarise key results with reference to study objectives | 9-10 |
| Limitations | 19 | Discuss limitations of the study, taking into account sources of potential bias or imprecision. Discuss both direction and magnitude of any potential bias | 11 |
| Interpretation | 20 | Give a cautious overall interpretation of results considering objectives, limitations, multiplicity of analyses, results from similar studies, and other relevant evidence | 11 |
| Generalisability | 21 | Discuss the generalisability (external validity) of the study results | 11 |
| Other information | | | |
| Funding | 22 | Give the source of funding and the role of the funders for the present study and, if applicable, for the original study on which the present article is based | 12 |

Reference:

von Elm E, Altman DG, Egger M, Pocock SJ, Gøtzsche PC, Vandenbroucke JP (2007) The Strengthening the Reporting of Observational Studies in Epidemiology (STROBE) statement: guidelines for reporting observational studies. Lancet 370(9596):1453-1457. <https://doi.org/10.1016/s0140-6736(07)61602-x>

**TABLE S2** A list of ICD 9-AM and ICD 10-AM diagnosis codes for RA.

| **ICD-9 AM** | **Description** | **ICD-10 AM** | **Description** |
| --- | --- | --- | --- |
| 714.0 | RA and other inflammatory polyarthropathies | M05.80- M05.89 | Seropositive (specified) with rheumatoid factor |
|  |  | M05.90- M05.99 | Seropositive (unspecified) |
|  |  | M06.00- M06.09 | Seronegative without rheumatoid factor |
|  |  | M06.20- M06.29 | Rheumatoid bursitis |
|  |  | M06.30- M06.39 | Rheumatoid nodule |
|  |  | M06.80- M06.89 | other specific, RA |
|  |  | M06.90- M06.99 | Unspecified, RA |
| 714.1 | Felty`s syndrome | M05.00- M05.09 | Felty`s syndrome |
| 714.2 | Other Rheumatoid Arthritis with visceral or systemic involvement | M05.20- M05.29 | Rheumatoid vasculitis |
| 714.3 | Juvenile chronic polyarthritis | M08.00- M08.09 | Juvenile RA with or without rheumatoid factor |
| 714.30 | Polyarticular juvenile RA, chronic or unspecified | M08.20- M08.29 | Juvenile RA with systemic onset, multiple sites |
|  |  | M08.90- M08.99 | Juvenile RA unspecified |
|  |  | M06.10- M06.19 | Still`s disease |
| 714.31 | Polyarticular juvenile RA, acute | M08.30- M08.39 | Juvenile RA polyarthritis (seronegative) |
| 714.32 | Pauciarticular juvenile RA | M08.40- M08.49 | Pauciarticular juvenile RA, unspecified |
| 714. 33 | Monoarticular juvenile RA | M08.80- M08.89 | Monoarticular juvenile RA |
| 714.4 | chronic postrheumatic arthropathy  Chronic rheumatoid nodular fibrositis  Jaccoud`s syndrome | M12.00- M12.09 | Chronic postrheumatic arthropathy [Jaccoud] |
| 714.8 | Other specified Inflammatory polyarthropathies | M6.40- M06.49 | Inflammatory polyarthropathy |
| 714.89 | Other (inflammatory polyarthropathy) |  |  |
| 714.9 | Unspecified inflammatory polyarthropathy |  |  |
| 714.81 | Interstitial lung disease | M05.10- M05.19 | Interstitial lung disease |

RA= Rheumatoid Arthritis, ICD-9 AM= standard International Classification of Disease, Ninth Revision, Australian Modification. ICD-10 AM= standard International Classification of Disease, Tenth Revision, Australian Modification.

**TABLE S3** Hospital separations for Rheumatoid Arthritis in Western Australia hospitals (1995-2014).

| **Year** | **Total RA hospital separations** | **WA hospital separations** | **RA per 1000 separations** | **RA separations per 1000 population** | **Other conditions** **separations** **per 1000 population** | **National separations per 1000 population** | **RA separations rate ratio** | **Other conditions separations rate ratio** | **Prevalence of RA percentage**  **(95% CI)** |
| --- | --- | --- | --- | --- | --- | --- | --- | --- | --- |
| 1995 | 3,518 | 451,195 | 7.8 | 2.03 | 257.9 | 259.9 | 0.008 | 0.992 | 0.78 (0.75- 0.81) |
| 1996 | 3,751 | 473,677 | 7.9 | 2.12 | 265.8 | 267.9 | 0.008 | 0.992 | 0.79 (0.77- 0.82) |
| 1997 | 3,994 | 506,721 | 7.9 | 2.22 | 279.6 | 281.8 | 0.008 | 0.992 | 0.78 (0.76- 0.81) |
| 1998 | 3,927 | 544,566 | 7.2 | 2.15 | 296.0 | 298.2 | 0.007 | 0.993 | 0.72 (0.70- 0.74) |
| 1999 | 2,785 | 575,489 | 4.8 | 1.50 | 308.9 | 310.4 | 0.005 | 0.995 | 0.48 (0.47- 0.50) |
| 2000 | 1,755 | 612,774 | 2.9 | 0.93 | 325.2 | 326.1 | 0.003 | 0.997 | 0.29 (0.27- 0.30) |
| 2001 | 1,577 | 617,891 | 2.6 | 0.83 | 323.3 | 324.1 | 0.003 | 0.997 | 0.26 (0.24- 0.27) |
| 2002 | 1,685 | 648,423 | 2.6 | 0.87 | 335.4 | 336.2 | 0.003 | 0.997 | 0.26 (0.25- 0.27) |
| 2003 | 1,908 | 657,439 | 2.9 | 0.98 | 335.7 | 336.7 | 0.003 | 0.997 | 0.29 (0.28- 0.30) |
| 2004 | 1,836 | 691,975 | 2.7 | 0.93 | 348.6 | 349.6 | 0.003 | 0.997 | 0.27 (0.25- 0.28) |
| 2005 | 2,045 | 714,353 | 2.9 | 1.02 | 354.2 | 355.2 | 0.003 | 0.997 | 0.29 (0.27- 0.30) |
| 2006 | 2,215 | 740,059 | 3.0 | 1.08 | 359.8 | 360.9 | 0.003 | 0.997 | 0.30 (0.29- 0.31) |
| 2007 | 2,282 | 783,620 | 2.9 | 1.08 | 371.0 | 372.1 | 0.003 | 0.997 | 0.29 (0.28- 0.30) |
| 2008 | 2,184 | 829,595 | 2.6 | 1.01 | 381.0 | 382.0 | 0.003 | 0.997 | 0.26 (0.25- 0.27) |
| 2009 | 2,682 | 887,209 | 3.0 | 1.20 | 394.8 | 396.0 | 0.003 | 0.997 | 0.30 (0.29- 0.31) |
| 2010 | 2,627 | 966,033 | 2.7 | 1.15 | 420.5 | 421.7 | 0.003 | 0.997 | 0.27 (0.26- 0.28) |
| 2011 | 2,597 | 1,024,462 | 2.5 | 1.10 | 434.2 | 435.3 | 0.003 | 0.997 | 0.25 (0.24- 0.26) |
| 2012 | 2,689 | 1,058,751 | 2.5 | 1.11 | 435.4 | 436.5 | 0.003 | 0.997 | 0.25 (0.24- 0.26) |
| 2013 | 2,281 | 1,070,399 | 2.1 | 0.92 | 429.5 | 430.4 | 0.002 | 0.998 | 0.21 (0.20- 0.22) |
| 2014 | 2,015 | 1,081,463 | 1.9 | 0.80 | 428.8 | 429.6 | 0.002 | 0.998 | 0.18 (0.17- 0.19) |

RA= Rheumatoid Arthritis, WA = Western Austral

**TABLE S4** The mean and confidence interval of rheumatoid arthritis patients' length of stays in

Western Australian hospitals (1995–2014).

| Characteristics | Male | | | | | | Female | | | |
| --- | --- | --- | --- | --- | --- | --- | --- | --- | --- | --- |
| Year of Hospital Separations | Mean | Upper 95% CI | | Lower 95% CI | | Total length of hospital stays | Mean | Upper 95% CI | Lower 95% CI | Total length of hospital stays |
| 1995 | 8.02 | | 8.66 | | 7.38 | 8,201 | 10.97 | 14.25 | 7.69 | 27,173 |
| 1996 | 8.56 | | 9.39 | | 7.73 | 8,793 | 9.52 | 10.09 | 8.95 | 25,721 |
| 1997 | 8.29 | | 9.01 | | 7.57 | 10,077 | 8.66 | 9.10 | 8.22 | 23,551 |
| 1998 | 8.79 | | 9.62 | | 7.96 | 10,169 | 8.48 | 8.99 | 7.97 | 23,065 |
| 1999 | 9.48 | | 10.38 | | 8.58 | 7,176 | 9.37 | 10.53 | 8.21 | 18,483 |
| 2000 | 8.78 | | 9.88 | | 7.68 | 4,256 | 10.29 | 11.11 | 9.47 | 12,582 |
| 2001 | 9.13 | | 10.48 | | 7.78 | 3,544 | 10.68 | 11.80 | 9.56 | 11,862 |
| 2002 | 10.85 | | 13.32 | | 8.38 | 3,927 | 8.90 | 9.71 | 8.09 | 10,384 |
| 2003 | 8.19 | | 11.01 | | 5.37 | 3,373 | 8.52 | 9.88 | 7.16 | 11,564 |
| 2004 | 7.81 | | 9.35 | | 6.27 | 3,251 | 7.73 | 8.51 | 6.95 | 9,956 |
| 2005 | 7.10 | | 8.59 | | 5.61 | 3,592 | 6.89 | 7.56 | 6.22 | 9,612 |
| 2006 | 6.04 | | 6.98 | | 5.10 | 3,546 | 6.64 | 7.24 | 6.04 | 9,788 |
| 2007 | 8.00 | | 12.83 | | 3.17 | 5,250 | 5.94 | 6.57 | 5.31 | 8,769 |
| 2008 | 4.84 | | 5.64 | | 4.04 | 2,698 | 4.79 | 5.30 | 4.28 | 7,012 |
| 2009 | 4.57 | | 5.35 | | 3.79 | 3,069 | 4.10 | 4.55 | 3.65 | 7,664 |
| 2010 | 4.28 | | 5.11 | | 3.45 | 2,860 | 3.76 | 4.21 | 3.31 | 6,750 |
| 2011 | 4.46 | | 5.48 | | 3.44 | 2,718 | 3.94 | 4.36 | 3.52 | 7,142 |
| 2012 | 3.53 | | 4.08 | | 2.98 | 2,258 | 3.98 | 4.48 | 3.48 | 7,500 |
| 2013 | 3.56 | | 4.28 | | 2.84 | 1,787 | 3.77 | 4.19 | 3.35 | 6,074 |
| 2014 | 5.23 | | 6.48 | | 3.98 | 2,417 | 3.75 | 4.14 | 3.36 | 5,514 |
| Total | - | | - | | - | 92,962 |  |  |  | 250,166 |

CI= confidence interval.

**Logistic Regression Analysis**

| **Model Summary - Frequent** | | | | | | | | | | | | | | | | | | | | | |
| --- | --- | --- | --- | --- | --- | --- | --- | --- | --- | --- | --- | --- | --- | --- | --- | --- | --- | --- | --- | --- | --- |
| **Model** | | **Deviance** | **AIC** | | **BIC** | | **df** | | **ΔΧ²** | | **p** | | **McFadden R²** | | **Nagelkerke R²** | | | **Tjur R²** | **Cox & Snell R²** | | |
| 1 |  | 54421.240 |  | 54423.240 |  | 54432.067 |  | 50352 |  |  |  |  |  | 0.000 |  |  |  | 0.000 |  |  |  |
| 2 |  | 54022.603 |  | 54036.603 |  | 54098.390 |  | 50346 |  | 398.637 |  | < .001 |  | 0.007 |  | 0.008 |  | 0.009 |  | 0.008 |  |
| 3 |  | 53956.737 |  | 53972.737 |  | 54043.352 |  | 50345 |  | 65.865 |  | < .001 |  | 0.009 |  | 0.009 |  | 0.010 |  | 0.009 |  |
| 4 |  | 53950.720 |  | 53970.720 |  | 54058.988 |  | 50343 |  | 6.017 |  | 0.049 |  | 0.009 |  | 0.009 |  | 0.010 |  | 0.009 |  |
| 5 |  | 53878.504 |  | 53922.504 |  | 54116.694 |  | 50331 |  | 72.216 |  | < .001 |  | 0.010 |  | 0.011 |  | 0.012 |  | 0.011 |  |
|  | | | | | | | | | | | | | | | | | | | | | |

| **Coefficients** | | | | | | | | | | | | | | | | | | | | | |
| --- | --- | --- | --- | --- | --- | --- | --- | --- | --- | --- | --- | --- | --- | --- | --- | --- | --- | --- | --- | --- | --- |
|  | | | | | | | | | | | | **Wald Test** | | | | | | **95% Confidence interval** | | | |
| **Model** | | **Parameter** | | **Estimate** | | **Standard Error** | | **Odds Ratio** | | **z** | | **Wald Statistic** | | **df** | | **p** | | **Lower bound** | | **Upper bound** | |
| 1 |  | (Intercept) |  | 1.203 |  | 0.011 |  | 3.330 |  | 113.770 |  | 12943.541 |  | 1 |  | < .001 |  | 1.182 |  | 1.224 |  |
| 2 |  | (Intercept) |  | -0.139 |  | 0.086 |  | 0.870 |  | -1.625 |  | 2.640 |  | 1 |  | 0.104 |  | -0.308 |  | 0.029 |  |
|  |  | 20- 29 years |  | 1.027 |  | 0.105 |  | 2.793 |  | 9.797 |  | 95.972 |  | 1 |  | < .001 |  | 0.822 |  | 1.232 |  |
|  |  | 30- 44 years |  | 1.263 |  | 0.091 |  | 3.537 |  | 13.810 |  | 190.727 |  | 1 |  | < .001 |  | 1.084 |  | 1.443 |  |
|  |  | 45- 59 years |  | 1.491 |  | 0.088 |  | 4.442 |  | 16.859 |  | 284.216 |  | 1 |  | < .001 |  | 1.318 |  | 1.664 |  |
|  |  | 60- 69 years |  | 1.489 |  | 0.089 |  | 4.431 |  | 16.770 |  | 281.230 |  | 1 |  | < .001 |  | 1.315 |  | 1.663 |  |
|  |  | 70- 84 years |  | 1.273 |  | 0.088 |  | 3.571 |  | 14.491 |  | 209.980 |  | 1 |  | < .001 |  | 1.101 |  | 1.445 |  |
|  |  | 85+ years |  | 1.117 |  | 0.096 |  | 3.055 |  | 11.605 |  | 134.686 |  | 1 |  | < .001 |  | 0.928 |  | 1.305 |  |
| 3 |  | (Intercept) |  | -0.281 |  | 0.088 |  | 0.755 |  | -3.204 |  | 10.264 |  | 1 |  | 0.001 |  | -0.452 |  | -0.109 |  |
|  |  | 20- 29 years |  | 1.018 |  | 0.105 |  | 2.766 |  | 9.698 |  | 94.045 |  | 1 |  | < .001 |  | 0.812 |  | 1.223 |  |
|  |  | 30- 44 years |  | 1.265 |  | 0.092 |  | 3.542 |  | 13.815 |  | 190.864 |  | 1 |  | < .001 |  | 1.085 |  | 1.444 |  |
|  |  | 45- 59 years |  | 1.495 |  | 0.089 |  | 4.459 |  | 16.886 |  | 285.128 |  | 1 |  | < .001 |  | 1.321 |  | 1.668 |  |
|  |  | 60- 69 years |  | 1.500 |  | 0.089 |  | 4.481 |  | 16.879 |  | 284.900 |  | 1 |  | < .001 |  | 1.326 |  | 1.674 |  |
|  |  | 70- 84 years |  | 1.278 |  | 0.088 |  | 3.589 |  | 14.535 |  | 211.274 |  | 1 |  | < .001 |  | 1.106 |  | 1.450 |  |
|  |  | 85+ years |  | 1.100 |  | 0.096 |  | 3.005 |  | 11.423 |  | 130.489 |  | 1 |  | < .001 |  | 0.911 |  | 1.289 |  |
|  |  | Female |  | 0.191 |  | 0.023 |  | 1.211 |  | 8.168 |  | 66.715 |  | 1 |  | < .001 |  | 0.146 |  | 0.237 |  |
| 4 |  | (Intercept) |  | -0.374 |  | 0.096 |  | 0.688 |  | -3.911 |  | 15.295 |  | 1 |  | < .001 |  | -0.561 |  | -0.187 |  |
|  |  | 20- 29 years |  | 1.011 |  | 0.105 |  | 2.747 |  | 9.627 |  | 92.673 |  | 1 |  | < .001 |  | 0.805 |  | 1.216 |  |
|  |  | 30- 44 years |  | 1.259 |  | 0.092 |  | 3.523 |  | 13.745 |  | 188.934 |  | 1 |  | < .001 |  | 1.080 |  | 1.439 |  |
|  |  | 45- 59 years |  | 1.493 |  | 0.089 |  | 4.451 |  | 16.863 |  | 284.361 |  | 1 |  | < .001 |  | 1.320 |  | 1.667 |  |
|  |  | 60- 69 years |  | 1.494 |  | 0.089 |  | 4.453 |  | 16.797 |  | 282.148 |  | 1 |  | < .001 |  | 1.319 |  | 1.668 |  |
|  |  | 70- 84 years |  | 1.269 |  | 0.088 |  | 3.557 |  | 14.416 |  | 207.832 |  | 1 |  | < .001 |  | 1.096 |  | 1.441 |  |
|  |  | 85+ years |  | 1.090 |  | 0.096 |  | 2.974 |  | 11.302 |  | 127.735 |  | 1 |  | < .001 |  | 0.901 |  | 1.279 |  |
|  |  | Female |  | 0.190 |  | 0.023 |  | 1.210 |  | 8.113 |  | 65.823 |  | 1 |  | < .001 |  | 0.144 |  | 0.236 |  |
|  |  | Major cities |  | 0.104 |  | 0.044 |  | 1.110 |  | 2.347 |  | 5.509 |  | 1 |  | 0.019 |  | 0.017 |  | 0.191 |  |
|  |  | Rural |  | 0.119 |  | 0.050 |  | 1.126 |  | 2.361 |  | 5.574 |  | 1 |  | 0.018 |  | 0.020 |  | 0.217 |  |
| 5 |  | (Intercept) |  | -0.047 |  | 0.245 |  | 0.954 |  | -0.191 |  | 0.037 |  | 1 |  | 0.848 |  | -0.528 |  | 0.434 |  |
|  |  | 20- 29 years |  | -0.089 |  | 0.342 |  | 0.915 |  | -0.261 |  | 0.068 |  | 1 |  | 0.794 |  | -0.760 |  | 0.582 |  |
|  |  | 30- 44 years |  | 0.419 |  | 0.270 |  | 1.521 |  | 1.553 |  | 2.412 |  | 1 |  | 0.120 |  | -0.110 |  | 0.948 |  |
|  |  | 45- 59 years |  | 1.310 |  | 0.254 |  | 3.707 |  | 5.156 |  | 26.581 |  | 1 |  | < .001 |  | 0.812 |  | 1.808 |  |
|  |  | 60- 69 years |  | 1.205 |  | 0.262 |  | 3.338 |  | 4.605 |  | 21.209 |  | 1 |  | < .001 |  | 0.692 |  | 1.719 |  |
|  |  | 70- 84 years |  | 1.023 |  | 0.265 |  | 2.780 |  | 3.856 |  | 14.871 |  | 1 |  | < .001 |  | 0.503 |  | 1.542 |  |
|  |  | 85+ years |  | 0.804 |  | 0.410 |  | 2.234 |  | 1.959 |  | 3.839 |  | 1 |  | 0.050 |  | -0.000 |  | 1.608 |  |
|  |  | Female |  | 0.187 |  | 0.024 |  | 1.205 |  | 7.950 |  | 63.210 |  | 1 |  | < .001 |  | 0.141 |  | 0.233 |  |
|  |  | Major cities |  | -0.254 |  | 0.265 |  | 0.776 |  | -0.957 |  | 0.916 |  | 1 |  | 0.339 |  | -0.774 |  | 0.266 |  |
|  |  | Rural |  | -0.297 |  | 0.319 |  | 0.743 |  | -0.931 |  | 0.866 |  | 1 |  | 0.352 |  | -0.922 |  | 0.328 |  |
|  |  | 20- 29 years * Major cities |  | 1.241 |  | 0.364 |  | 3.460 |  | 3.409 |  | 11.619 |  | 1 |  | < .001 |  | 0.528 |  | 1.955 |  |
|  |  | 30- 44 years * Major cities |  | 0.980 |  | 0.291 |  | 2.665 |  | 3.367 |  | 11.338 |  | 1 |  | < .001 |  | 0.410 |  | 1.551 |  |
|  |  | 45- 59 years * Major cities |  | 0.179 |  | 0.275 |  | 1.196 |  | 0.650 |  | 0.422 |  | 1 |  | 0.516 |  | -0.361 |  | 0.718 |  |
|  |  | 60- 69 years * Major cities |  | 0.317 |  | 0.282 |  | 1.373 |  | 1.122 |  | 1.258 |  | 1 |  | 0.262 |  | -0.237 |  | 0.870 |  |
|  |  | 70- 84 years * Major cities |  | 0.264 |  | 0.285 |  | 1.302 |  | 0.925 |  | 0.856 |  | 1 |  | 0.355 |  | -0.295 |  | 0.823 |  |
|  |  | 85+ years * Major cities |  | 0.327 |  | 0.425 |  | 1.387 |  | 0.768 |  | 0.590 |  | 1 |  | 0.442 |  | -0.507 |  | 1.161 |  |
|  |  | 20- 29 years * Rural |  | 0.988 |  | 0.424 |  | 2.686 |  | 2.333 |  | 5.442 |  | 1 |  | 0.020 |  | 0.158 |  | 1.818 |  |
|  |  | 30- 44 years * Rural |  | 0.612 |  | 0.348 |  | 1.844 |  | 1.757 |  | 3.087 |  | 1 |  | 0.079 |  | -0.071 |  | 1.294 |  |
|  |  | 45- 59 years * Rural |  | 0.353 |  | 0.331 |  | 1.423 |  | 1.067 |  | 1.138 |  | 1 |  | 0.286 |  | -0.295 |  | 1.001 |  |
|  |  | 60- 69 years * Rural |  | 0.390 |  | 0.337 |  | 1.477 |  | 1.157 |  | 1.338 |  | 1 |  | 0.247 |  | -0.271 |  | 1.051 |  |
|  |  | 70- 84 years * Rural |  | 0.401 |  | 0.338 |  | 1.493 |  | 1.186 |  | 1.406 |  | 1 |  | 0.236 |  | -0.262 |  | 1.064 |  |
|  |  | 85+ years * Rural |  | 0.331 |  | 0.474 |  | 1.392 |  | 0.698 |  | 0.487 |  | 1 |  | 0.485 |  | -0.598 |  | 1.260 |  |
|  | | | | | | | | | | | | | | | | | | | | | |
| *Note.*  Frequent level '1' coded as class 1. | | | | | | | | | | | | | | | | | | | | | |

Call:

glm(formula = Frequent ~ female:old:rural, family = binomial(link = "logit"),

data = mydata)

Deviance Residuals:

Min 1Q Median 3Q Max

-1.8402 0.6376 0.7268 0.7268 0.7268

Coefficients:

Estimate Std. Error z value Pr(>|z|)

(Intercept) 1.19637 0.01069 111.930 < 2e-16 ***

female:old:rural 0.29353 0.07393 3.971 7.17e-05 ***

---

Signif. codes: 0 ‘***’ 0.001 ‘**’ 0.01 ‘*’ 0.05 ‘.’ 0.1 ‘ ’ 1

(Dispersion parameter for binomial family taken to be 1)

Null deviance: 54421 on 50352 degrees of freedom

Residual deviance: 54405 on 50351 degrees of freedom

AIC: 54409

Number of Fisher Scoring iterations: 4

(Intercept) female:old:rural

3.308097 1.341158

2.5 % 97.5 %

(Intercept) 3.239642 3.378264

female:old:rural 1.162639 1.553673

**Table S5** Odd interaction ratio between female rheumatoid arthritis patients who were 60-69 years old and lived in rural areas.

|  | | B | S.E. | Wald | df | Sig. | Exp(B) | 95% C.I.for EXP(B) | |
| --- | --- | --- | --- | --- | --- | --- | --- | --- | --- |
|  |  |  |  |  |  |  |  | Lower | Upper |
| Step 1^a^ | Female(1) by old(1) by rural(1) | .294 | .074 | 15.765 | 1 | <.001 | 1.341 | 1.160 | 1.550 |
|  | Constant | 1.196 | .011 | 12528.320 | 1 | .000 | 3.308 |  |  |
| a. Variable(s) entered on step 1: female * old * rural. | | | | | | | | | |

***Regression Model with Segmented Relationship(s)***

Call:

segmented.lm(obj = fit, seg.Z = ~Year, psi = 2003)

Estimated Break-Point(s):

Est. St.Err

psi1.Year 2001 0.674

Meaningful coefficients of the linear terms:

Estimate Std. Error t value Pr(>|t|)

(Intercept) 198.68872 28.69394 6.924 3.42e-06 ***

Year -0.09915 0.01436 -6.902 3.56e-06 ***

U1.Year 0.09447 0.01491 6.337 NA

---

Signif. codes: 0 ‘***’ 0.001 ‘**’ 0.01 ‘*’ 0.05 ‘.’ 0.1 ‘ ’ 1

Residual standard error: 0.06009 on 16 degrees of freedom

Multiple R-Squared: 0.9306, Adjusted R-squared: 0.9176

Boot restarting based on 6 samples. Last fit:

Convergence attained in 2 iterations (rel. change 2.5422e-08)

***Regression Model with Segmented Relationship(s)***

Call:

segmented.lm(obj = fit, seg.Z = ~Year, psi = 2001)

Estimated Break-Point(s):

Est. St.Err

psi1.Year 2001 0.674

Meaningful coefficients of the linear terms:

Estimate Std. Error t value Pr(>|t|)

(Intercept) 198.68872 28.69394 6.924 3.42e-06 ***

Year -0.09915 0.01436 -6.902 3.56e-06 ***

U1.Year 0.09447 0.01491 6.337 NA

---

Signif. codes: 0 ‘***’ 0.001 ‘**’ 0.01 ‘*’ 0.05 ‘.’ 0.1 ‘ ’ 1

Residual standard error: 0.06009 on 16 degrees of freedom

Multiple R-Squared: 0.9306, Adjusted R-squared: 0.9176

Boot restarting based on 6 samples. Last fit:

Convergence attained in 2 iterations (rel. change 2.5422e-08)


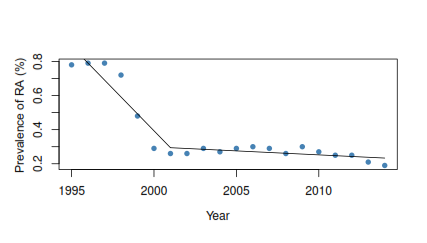


Figure S1: Joinpoint regression model visualisation.

RA= Rheumatoid Arthritis. *Error bars indicate 95% confidence interval.

Figure S2: The prevalence of Rheumatoid Arthritis percentage in Western Australia extrapolated from biological therapy usage data over time.

**TABLE S6** Total RA bDMARDs utilisation (DDD/1000 population/day) and number RA patients use standard dose daily (DDD) of RA bDMARDs at WA, 1995–2014.

| **Year** | **Total RA bDMARDs utilisation (DDD/1000 population/day)** | **WA general population** | **Number of RA patients use standard dose daily of DMARDs at WA** | **Prevalence of RA bDMARDs use per 1000 population** |
| --- | --- | --- | --- | --- |
| 2003 | 0.01 | 1,952,741 | 14 | 0.0071 (95% CI: 0.0039- 0.0120) |
| 2004 | 0.08 | 1,979,542 | 158 | 0.0798 (95% CI: 0.0678- 0.0932) |
| 2005 | 0.16 | 2,011,207 | 329 | 0.1635 (95% CI: 0.1463- 0.1822) |
| 2006 | 0.23 | 2,050,581 | 476 | 0.2321 (95% CI: 0.2117- 0.2539) |
| 2007 | 0.31 | 2,106,139 | 643 | 0.3052 (95% CI: 0.2821- 0.3298) |
| 2008 | 0.50 | 2,171,700 | 1,094 | 0.5037 (95% CI: 0.4743- 0.5345) |
| 2009 | 0.60 | 2,240,250 | 1,338 | 0.5972 (95% CI: 0.5656- 0.6301) |
| 2010 | 0.59 | 2,290,845 | 1,361 | 0.5941 (95% CI: 0.5629- 0.6265) |
| 2011 | 0.63 | 2,353,409 | 1,475 | 0.6267 (95% CI: 0.5951- 0.6595) |
| 2012 | 0.77 | 2,425,507 | 1,859 | 0.7664 (95% CI: 0.7320- 0.8020) |
| 2013 | 0.66 | 2,486,944 | 1,649 | 0.6630 (95% CI: 0.6314- 0.6958) |
| 2014 | 1.00 | 2,517,608 | 2,510 | 0.9969 (95% CI: 0.9583- 1.0367) |

Abbreviations: bDMARDs, biologic disease-modifying anti-rheumatic drugs included Abatacept, Adalimumab, Certolizumab, Etanercept, Golimumab, Infliximab, Rituximab, Tocilizumab; DDD, defined daily doses; RA, Rheumatoid arthritis; WA, Western Australia, 95% CI, 95% Confidence Interval.

**Appendix A: Ethics approval letter.**


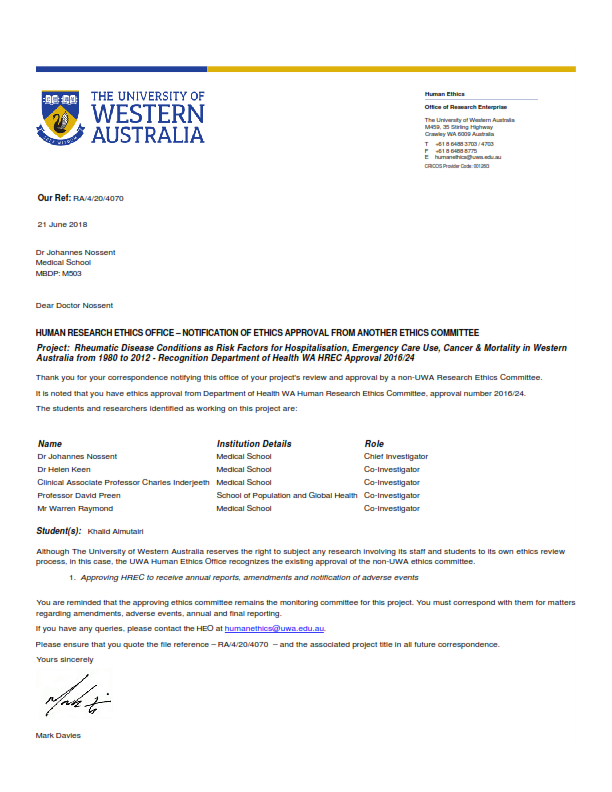

Supplement: Supplementary file 1 — Additional file 1. Table S1: STROBE Statement checklist of items that should be included in reports of observational studies. Table S2: A list of ICD 9-AM and ICD 10-AM diagnosis codes for RA. Table S3: Hospital separations for Rheumatoid Arthritis in Western Australia hospitals (1995-2014). Table S4: The mean and confidence interval of rheumatoid arthritis patients' length of stays in Western Australian hospitals (1995–2014). Table S5: Odd interaction ratio between female rheumatoid arthritis patients who were 60-69 years old and lived in rural areas. Figure S1: Joinpoint regression model visualisation. Figure S2: The prevalence of Rheumatoid Arthritis percentage in Western Australia extrapolated from biological therapy usage data over time. Table S6: Total RA bDMARDs utilisation (DDD/1000 population/day) and number RA patients use standard dose daily (DDD) of RA bDMARDs at WA, 1995–2014. Appendix A: Ethics approval letter. [file 41927_2022_324_MOESM1_ESM.docx]
